# Microvascular Complications in Adolescents with Type 1 Diabetes Mellitus

**DOI:** 10.4274/Jcrpe.994

**Published:** 2013-09-18

**Authors:** Fatma Demirel, Derya Tepe, Özlem Kara, İhsan Esen

**Affiliations:** 1 Ankara Pediatric Hematology and Oncology Training Hospital, Department of Pediatric Endocrinology, Ankara, Turkey

**Keywords:** type 1 diabetes mellitus, adolescent, complication, screening, consensus

## Abstract

**Objective:** Screening of complications is an important part of diabetes care. The aim of this study was to investigate diabetic complications and related risk factors in adolescents with type 1 diabetes mellitus (T1DM).

**Methods:** This cross-sectional study was conducted on type 1 diabetics who were over 11 years of age or had a diabetes duration of 2 years and included 155 adolescents with T1DM (67 male, 88 female). The mean age of the patients was 14.4±2.1 years. Mean diabetes duration was 6.3±2.9 years. The patients were screened for diabetic nephropathy, retinopathy and peripheral neuropathy.

**Results:** Mean glycosylated hemoglobin (HbA1c) level of the study group was 8.4%. The frequency of microalbuminuria and peripheral neuropathy were 16.1% and 0.6%, respectively. None of the patients had diabetic retinopathy. Dyslipidemia and hypertension rates were 30.3% and 12.3%, respectively. Risk factors associated with microalbuminuria were hypertension, higher HbA1c levels, longer diabetes duration and dyslipidemia.

**Conclusion:** Early diagnosis and treatment of hypertension and dyslipidemia as well as achieving a better metabolic control are important in prevention or postponement of complications in patients with T1DM. Yearly screening for diabetic nephropathy should be started 2 years after the onset of the diabetes.

**Conflict of interest:**None declared.

## INTRODUCTION

Type 1 diabetes mellitus (T1DM) is the most common chronic disease in childhood. Good metabolic control during childhood and adolescence is crucial for the future health and life quality of these patients (1,2). Long-term micro- and macrovascular complications are challenging health problems which affect both quality of life and life expectancy in diabetic patients. There are studies in which hypertension, microalbuminuria, retinopathy, and neuropathy are reported with variable frequencies related to metabolic control level and diabetes duration in adolescents with T1DM (3,4,5,6). It is noteworthy that especially in adolescents, microvascular complication frequency is more than the expected in 2-5 years’ time following initiation of T1DM (1). In the latest global consensus report published in 2011 by the International Society for Pediatric and Adolescent Diabetes (ISPAD) and International Diabetes Foundation (IDF), it has been recommended that annual screening for microvascular complications should be started from age 11 years and after two years diabetes duration (7). Periodical screening for complications in diabetic patients, by providing information both to the diabetes team and the family, serves to improve metabolic control, and consequently early precautions can be taken. In this present study, screening results for microvascular complications are evaluated according to current consensus recommendations in T1DM patients of a pediatric endocrinology clinic, aiming to define the time of occurrence of complications and the related risk factors involved. 

## METHODS

The study was designed as a cross-sectional analysis covering data from 2011 to 2012 on adolescent T1DM patients followed in the Pediatric Endocrinology Clinic of the Ankara Pediatric Hematology Oncology Training and Research Hospital. The study group consisted of 155 T1DM subjects (67 male, 88 female) diagnosed in their prepubertal period, who were over age 11 years at the time of the study and had a diabetes duration of longer than 2 years. All patients were seen at 3-month intervals and all were on multiple dose flexible insulin treatment. Chronological age, age at diagnosis, diabetes duration, height, weight, body mass index (BMI), and in cases with microvascular complications, date of diagnosis of these complication and diabetes duration at that time, were recorded. Patients with T2DM, MODY, and syndromic diabetes (Down syndrome, Wolfram syndrome, etc.) were excluded from the study. Informed consent was taken from the patients and their families. The study was approved by the local ethics committee of the hospital.

**Screening for Microvascular Complications**

In screening for diabetic nephropathy, urine microalbumin level and renal ultrasonography were requested. A urinary albumin excretion of 30-300 mg/L in at least two urine samples in a 24-hour period was evaluated as microalbuminuria, and an albumin level over 300 mg/L in the urine was defined as macroalbuminuria (7).

For detection of retinopathy, fundus examination was performed by an ophthalmologist, after mydriatic application. Fundus imaging was performed in suspected cases.

For evaluation of neuropathy, a neurological examination was performed by a pediatric neurologist and in suspected cases, electromyography was performed.

**Assessment of Hypertension**

Systolic and diastolic blood pressure percentiles defined for Turkish children according to age and gender were used for evaluation of blood pressure (8). A blood pressure value over 95% of the reference value for age and gender in at least two measurements taken after a 10-minute resting period by a standard aneroid manometer device was accepted to be indicative of hypertension. Hypertension was definitely diagnosed in these cases by performing further evaluation via 24-hour Holter monitoring. Previously published data were used to define hypertension by 24-hour blood pressure monitoring (9). In patients with co-existent microalbuminuria, the patient records were investigated for presence of hypertension prior to the microalbuminuria.

**Assessment of Metabolic Control**

Mean value of the past year’s glycosylated hemoglobin (HbA1c) was used for assessment of metabolic control. In cases with complications, the mean HbA1c value in the year preceding the date of the defined complication was used in the assessment.

**Evaluation of Dyslipidemia**

Blood levels of total cholesterol (TC), triglyceride (TG), and high-density lipoprotein cholesterol (HDL-C) were measured in the biochemistry laboratory of our hospital with standard methods using a Roche Modular-P device. Low-density lipoprotein cholesterol (LDL-C) values were computed according to the Friedewald formula. In accordance with the recommendations of American Diabetes Association and, National Cholesterol Education Program dyslipidemia was defined as a TC level >200 mg/dL, a TG level >150 mg/dL, a LDL-C level >130 mg/dL, and a HDL-C level <40 mg/dL (10). For a better evaluation of dyslipidemia as a risk factor in the development of complications, the mean figure for lipid values at two different periods of the year at which the complication was detected was calculated. In cases without complication, the most recent lipid results were recorded.

**Statistical Analysis**

Statistical analysis was performed using SPSS version 13.0 (SPSS Inc., Chicago, IL). The data were presented as means±standard deviation (SD), where appropriate. A p-value of less than 0.05 was accepted as significant. Mann-Whitney U and chi-square tests were used for comparison of continuous and categorical data, respectively. Binary logistic regression analysis was used to examine the association between microalbuminuria and explanatory variables (hypertension, HbA1c level, diabetes duration, dyslipidemia, etc). Each variable was assessed individually step by step in the regression model. Those which were statistically significant were included in the final model.

## RESULTS

Mean diabetes duration of the study patients was 6.3±2.9 (2-16) years. Diabetes duration was 2.0-4.9 years in 27.6% (n=43), 5.0-9.9 years in 58.2% (n=90), and 10 years and more in 14.2% (n=22) of the cases. All cases were in their adolescent ages, and 78.7% (n=122) of them had been diagnosed in their prepubertal period. Clinical and laboratory characteristics of the male and female patients are shown in Table 1.

In our study group of diabetic patients, 25 cases (16.1%) were found to have microalbuminuria. One patient also developed peripheral neuropathy. There were no cases with retinopathy. When patients with and without microalbuminuria were compared, significant differences were observed in age at initial diagnosis, diabetes duration, HbA1c level, hypertension, and dyslipidemia frequencies between the two groups (Table 2).

High blood pressure, high HbA1c levels, long diabetes duration, and dyslipidemia were all found to be significant risk factors for microalbuminuria development (Table 3).

Time of initial diagnosis of microalbuminuria after diagnosis and time of initiation of ACE inhibitor (enalapril) treatment ranged between 10 months and 13.1 years (mean 5.3 years). Of these cases, five (24%) developed this complication within the first 2 years of the disease, whereas 6 patients (28%) developed complications in 2-5 years. Out of 25 cases with microalbuminuria, 9 had hypertension; 8 of these patients were previously diagnosed with hypertension, whereas 1 case was diagnosed with hypertension concomitantly with microalbuminuria. ACE inhibitors were prescribed to all hypertensive cases as soon as this complication was detected. However, it was found that compliance to this treatment was poor and that the patients did not use ACE inhibitors regularly until they developed microalbuminuria. Only one male patient reported that he had been smoking in the past one year. No microvascular complications or hypertension were detected in this patient. 

## DISCUSSION

Microalbuminuria was the most common microvascular complication in this group of adolescents with T1DM and it was present in 16.1% of the cases. Microalbuminuria was to be related to higher HbA1c levels, dyslipidemia, hypertension, longer diabetes duration, and to younger age at diagnosis. The most prominent risk factors for microalbuminuria were hypertension, higher HbA1c levels, longer diabetes duration, and dyslipidemia. Half of the 25 patients with microvascular complications were detected to have persistent microalbuminuria in the first 5 years of diagnosis, and approximately in one quarter of these, the microalbuminuria was diagnosed in their first 2 years of diabetes.

Diabetic nephropathy is the most frequently observed microvascular complication in adolescents with T1DM and the one with the earliest onset. In earlier studies, microalbuminuria rates were reported to vary between 3% and 25.4%, depending on diabetes duration (3,4,5,11,12,13). In adolescent diabetics, macroalbuminuria, end-stage renal disease (ESRD), retinopathy, and neuropathy were also reported with various frequencies (3,4,5,11,13). In our study group, there were no cases with macroalbuminuria or ESRD. Since the follow-ups of our patients were performed regularly at 3-month intervals, we believe that the detection and treatment of microalbuminuria at an early stage might have had a role in preventing macroalbuminuria and ESRD development. Peripheral neuropathy was observed in one case, and there were no cases with retinopathy. These findings can be attributed to the relatively short diabetes duration as well as to the lower HbA1c values of our patients as compared with those in other studies.

In our study group, hypertension was detected in 19 cases, and 1/3 of cases with microalbuminuria were hypertensive. In the risk analysis, hypertension was defined as the most significant risk factor for microalbuminuria. Similar outcomes were reported in both adolescent and adult screening studies for diabetic complications (4,13,14). Hypertension was found to increase cardiovascular risk in T1DM patients, and to expedite ESRD progression in cases with microalbuminuria (15,16). We believe that blood pressure measurements should be a part of routine examinations in outpatient visits scheduled at 3-month intervals for all T1DM patients starting from the time of diagnosis. Treatment with ACE inhibitors in hypertensive cases and close monitoring of this treatment would contribute to delayed development of microvascular complications.

The effect of good metabolic control on prevention and delay of microvascular complications has been well established by the Diabetes Control and Complications Trial (DCCT) results (17). In the adolescent cohort of this study, it was shown that a difference of 1.7% in intensive treatment and HbA1c led to a decrease of more than 50% in frequency of retinopathy, neuropathy, and microalbuminuria (18). In the Epidemiology of Diabetes Interventions and Complications (EDIC) study, the maintaining of this effect was also defined (19). In many studies, significantly higher HbA1c levels were shown in diabetic cases with microalbuminuria (3,4,5,11,12,13,14,15). Our results also demonstrate the reported relationship between poor glycemic control and microalbuminuria. It is therefore very important to achieve the best possible glycemic control in childhood and adolescence to prevent or to delay long term micro and macrovascular complications of T1DM.

The relationship of dyslipidemia with diabetic nephropathy and retinopathy was revealed by DCCT/EDIC studies (20,21). In one study, it was shown that increased cholesterol in serum increased the formation of advanced glycolysation end products (AGE) and that these products were related to diabetic renal disease (22). In our study, we found that dyslipidemia prevalence was significantly high in patients with microalbuminuria and that dyslipidemia was an independent risk factor for diabetic nephropathy.

Longer duration of diabetes, older age and puberty were determined as risk factors for complications in previous reports (4,11,13). For the same diabetes duration, age and puberty increase the risk for retinopathy and elevated albumin excretion rate (5). Changes in nutrition habits and life style in puberty, together with hormonal and metabolic changes which increase insulin resistance, complicate achievement of glycemic control. High blood glucose level and high HbA1c values contribute to the development of microvascular complications. In our study, all cases were adolescents, and there were no significant age differences between the groups with and without microvascular complications. Similar to previous studies, we also determined that longer diabetes duration was related to microalbuminuria rates (4,11,13).

In recent publications, it has been reported that male gender, smoking and high BMI were additional risk factors for the development of diabetic nephropathy (12,13,16,23,24). In our study, no difference in frequency of microalbuminuria was observed between males and females. Although obesity was slightly more frequent in girls than in boys, no difference in microalbuminuria was detected between obese and non-obese subjects. Fortunately, smoking frequency was very low in our diabetic cases. In a large cohort of German and Austrian adolescents and young adults, smoking frequency was reported as 10.5% and 34.8%, respectively (16). Because smoking is very significantly related to hypertension, diabetic nephropathy and cardiovascular risk factors, the harmful effects of smoking should be an important part of the health advice offered in the follow-up visits of children and adolescents with T1DM (6,25,26).

We believe that one important result of our study was to show that microalbuminuria can develop a short time after the onset of diabetes. Five of our patients developed microalbuminuria within their first 2 years of diabetes, and 12 patients within their first 5 years. The patients who developed microalbuminuria within their first 2 years of diabetes were found to have proteinuria in their spot urine samples during their routine follow-up visits and presence of microalbuminuria was definitely diagnosed in the 24-hour urine samples of two of these patients. We recommend that microalbuminuria screening should be started after the 2nd year of the diagnosis of diabetes and also wish to emphasize that proteinuria detected in spot urine samples should also be considered significant during routine controls.

The weakest aspect of our study is that it was a single-centered, cross-sectional study, and therefore included a relatively small number of microalbuminuria cases, making generalizations difficult. However, we believe that our results have provided significant data to review the rationality of the current consensus on screening.

Screening for microvascular complications in adolescents with T1DM is an appropriate approach when the second year of diagnosis is completed, or after 11 years of age, as has been indicated in the ISPAD/IDF consensus for microalbuminuria. However, it should also be remembered that diabetic nephropathy can have an earlier start in some cases. Our results and those of the Australian screening study indicate that microvascular complications other than nephropathy are very rare in the early period (3). Therefore, it might be more appropriate to decide on the screening time for diabetic retinopathy and peripheral neuropathy according to the findings of individual patients. Patients with poor metabolic control, with dyslipidemia and hypertension can be included in the early screening program, while this can be postponed to a visit after 5 years of diabetes in cases with good metabolic control and without hypertension or dyslipidemia.

In conclusion, in adolescents with T1DM, diabetic nephropathy is not rare in the early phase of the diabetes. Starting the screening after the first 2 years of the diabetes appears to be an appropriate approach. In addition to good metabolic control to prevent and to delay microvascular complications in diabetic children and adolescents, early diagnosis and treatment of hypertension and dyslipidemia are also important.

## Figures and Tables

**Table 1:**
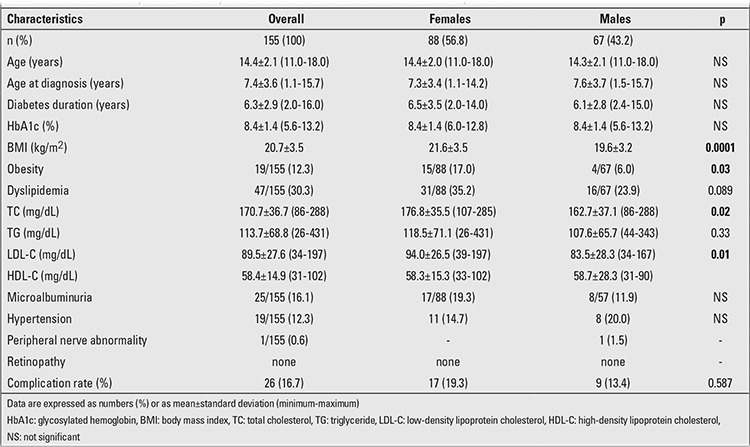
Characteristics of patients with >2 years diabetes duration assessed for diabetic complications

**Table 2:**
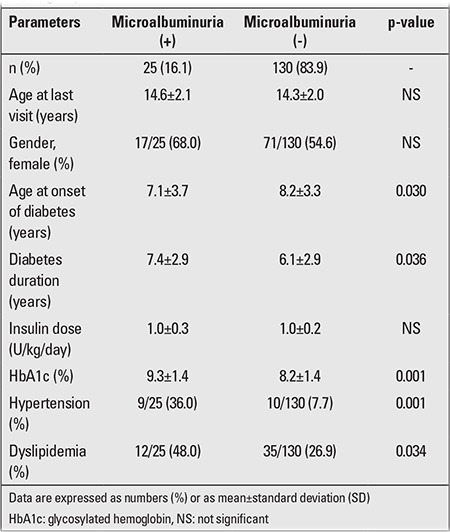
Clinical and laboratory differences between microalbuminuria (+)/(-) groups

**Table 3:**
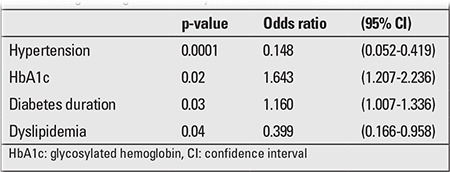
Logistic regression analysis of risk factors for microalbuminuria
